# Comprehensive Characterization of the Molecular Structure and Properties of Pitch-like Products from Coal Dissolution at Mild Temperature Using Heavy Solvents of Coal and Petroleum Origin

**DOI:** 10.3390/ma18071660

**Published:** 2025-04-04

**Authors:** Peter Kuznetsov, Budeebazar Avid, Ludmila Kuznetsova, Xing Fan, Jian-Fang Xu, Evgeniy Kamenskiy, Sergey Lyrschikov

**Affiliations:** 1 Institute of Chemistry and Chemical Technology SB RAS, Federal Research Center “Krasnoyarsk Science Center SBRAS”, 50-24 Akademgorodok, Krasnoyarsk 660036, Russia; drandmalada@gmail.com (L.K.); kamenskiy1986@mail.ru (E.K.); 2 Institute of Chemistry and Chemical Technology, Mongolian Academy of Sciences, Ave. Enkhtaivan, 54b, Ulaanbaatar 13330, Mongolia; avidmas@gmail.com; 3 State Key Laboratory of Chemistry and Utilization of Carbon-Based Energy Resources, School of Chemical Engineering and Technology, Xinjiang University, Urumqi 830017, China; fanxing@sdust.edu.cn (X.F.); xjf@stu.xju.edu.cn (J.-F.X.); 4 Institute of Coal Chemistry and Material Science SB RAS, FRC CCC SB RAS, 18 Sovetskij Ave., Kemerovo 650000, Russia; serstud@mail.ru

**Keywords:** coal, solvents, dissolution, pitch, aromaticity, needle coke

## Abstract

The chemical composition and molecular structure of the pitch-like products obtained by liquid-phase reaction of bituminous coal with heavy hydrocarbon fractions of coal and petroleum origin as solvents at a moderate temperature were comprehensively characterized in terms of a new aromatic feedstock for needle coke and other valuable high-tech carbon materials. The molecular parameters of the products were characterized by using FTIR, ^1^H NMR, ^13^C NMR and XPS. Liquid-phase chromatography was used to analyze benzo(a)pyrene (BaP) as a carcinogenicity marker. The chemical composition and the characteristics of the molecular structure of the products were shown to depend greatly on the solvent used. The product obtained using coal tar as a solvent was highly aromatic, its polyaromatic nuclei consisted predominantly of protonated and pericondensed cycles sparsely substituted by CH_3_ and occasionally CH_2_ groups. The product obtained using petroleum-derived heavy gas oil as solvent was much less aromatic and prone to autogenous surface oxidation. Its aromatic nuclei contained mainly protonated and highly alkylated catacondensed chains. The intermediate structural parameters were characteristic of the product obtained using binary solvent. A remarkable feature of the pitch-like products obtained was a reduced BaP concentration (up to 40 times compared to typical coal-tar pitch). In terms of the molecular structure, the pitch-like products obtained by low-temperature dissolution of coal can serve as a new polyaromatic feedstock with a reduced carcinogenicity for the production of valuable high-tech carbon materials, needle coke, in particular.

## 1. Introduction

Needle coke is an anisotropic carbon material with excellent electrical conductivity, thermal stability, oxidation resistance, and high graphitizability [1,2]. Due to these unique properties, needle coke is a main raw material for the production of a wide range of graphite materials for the most important fields of engineering and technology: for production of various graphite electrodes [2] and modern energy devices for storing and using energy, such as lithium-ion batteries and supercapacitors [3], graphite rods for nuclear reactors, various aerospace engineering products. By far the most important and large-scale needle coke application is in the production of ultra-high-power graphite electrodes for smelting of high-quality steel in the electric arc furnaces [2]. This method is intensively progressing throughout the world because of its efficiency, high quality of steel produced and favorable environmental characteristics [4,5].

Needle coke is produced by delayed carbonization of the aromatic raw materials [1,6]. During its preparation, the components of the aromatic matter go through the following stages as temperature increases: aromatic precursor → anisotropic mesophase pitch → “green needle coke” → calcined needle coke. The liquid-phase stage of the process includes the formation from the aromatic molecules of mesophase spherules, their coalescence and the oriented development of an anisotropic bulk mesophase-the precursor of needle coke. The main raw materials currently used in needle coke production are coal tar and residual fractions of primary and secondary oil refining. They are subjected to specific upgrading to meet compositional requirements. The main requirements are high content of aromatic hydrocarbons (aromaticity of 60–85%), moderate asphaltene content, low sulfur (preferably less than 1 wt.%), metals and ash, and high initial boiling point (more than 250 °C) [7,8]. The aromatic index is the key role in the high-quality needle coke production. Critically important components are planar polyaromatic hydrocarbons of moderate chemical reactivity, having certain configuration with three to five rings with optimal degree of substitution. Favorable components of the feedstock are also naphthenic and naphthenic-aromatic hydrocarbons. They ensure controlled mesophase development due to hydrogen donation ability. Multisubstituted aromatics demotes coalescence of mesophase spherules and anisotropy of the resultant coke.

It should be noted that, currently, the entire global petroleum industry is characterized by a steady increase in the share of high-sulfur heavy petroleum [9,10]. Its use requires complex purification from sulfur, asphaltenes, metals and aromatization, which significantly increases the cost of the final product. The resources of coal tar (coal-tar pitch) are also limited, its production volume has been steadily declining in recent decades [11,12] because of irreversible decline in demand for metallurgical coke [13]. Also, coal tar has a disadvantage associated with a high content of environmentally hazardous carcinogenic compounds, such as benzo(a)pyrene. Growing demand for polyaromatic feedstock for the production of many high value-added carbon materials is driving the search for new feedstock.

A promising and reliable polyaromatic feedstock can be the products of chemical processing of coal since it is an inherently polyaromatic matter with abundant reserves globally. However, the polyaromatic fragments in the coal are bond in a cross-linked polymer-like structure. Coal dissolution under mild conditions is considered [14,15,16,17] as a key strategy of breaking down the cross-linked polymer-like coal structure into polyaromatic molecules. Of recent, this process has attracted much attention, efforts are made to study the macromolecular structure of coal in detail and mechanism of its conversion into soluble substances [18,19,20].

The authors [21] reported that the polyaromatic residual fraction from the commercial coal liquefaction process at the Shenhua plant in China is a high-quality feedstock for the production of needle coke. Shui et al. [22] conducted dissolution of coals using 1-methylnaphthalene (1-MN) and crude methylnaphthalene oil (CMN) as solvents to obtain soluble products. FTIR and CP/MAS ^13^C NMR spectra showed the soluble substances obtained to consist of predominantly aromatics (the aromaticity of 60 to 80% depending on the solvent used). More aromatic substances were produced from coal dissolution using CMN as solvent. In a recent paper [23], the authors demonstrated all the soluble products derived from coals to show better caking property, lower softening temperatures, and greater caking indexes than the commonly used fat coals. Griffith et al. [24] used light cycle oil from the catalytic cracking of vacuum gas oil as solvent for coal dissolution at mild temperature of 350 °C. The products obtained from the Pittsburgh bituminous coal were shown to consist of bi- and tricyclic aromatic compounds. Rahman et al. [14] studied the composition of the products obtained by coal dissolution using heavy hydrocarbon fractions as solvents. The predominantly aromatic nature of the products was shown by FTIR and CP/MAS ^13^C NMR techniques.

The researchers [25,26,27,28] obtained ash-free HyperCoal product by coal dissolution at near 380–400 °C using recycle two-ring aromatics as solvents. HyperCoal product has gained increasing attention due to excellent properties (low softening point, high calorific value, good reaction performance and excellent thermoplasticity). It was demonstrated that HyperCoal is an effective feedstock for carbon binders and functional carbon materials, such as anodes [28] and carbon fibers [26,27] with high tensile strength (of 1.83 to 3.00 GPa).

Researchers [29,30] investigated the dissolution of bituminous coals at 400–420 °C using anthracene oil as a recycle solvent. The pitch-like products obtained were intended for the production of high-quality carbon anodes. In recent article [31], the authors reported the results of mild coal dissolutiom using catalytic cracking decant oil as solvent. It was demonstrated that the mesophase pitch derived from the coal extract can be used to obtain carbon fibers with graphitic texture, high modulus and tensile strength. Also, they reported for the first time that coal wastes from the coal preparation plant can be utilized to obtain aromatic solubles [32]. It was noted that this finding makes it possible to remediate waste coal impoundments and to obtain low-cost coal-based fibers.

The authors [33,34,35] conducted a comparative study of the molecular structures of different pitches and their ability to form mesophase and needle coke. Vacuum petroleum residue, coal tar and coal dissolution product were used as the raw materials for the preparation of VRP, CTP and CDP pitches, respectively. The latter were studied in detail using a set of various analytical techniques, such as GC-MS, ^1^H and ^13^C NMR and Raman spectroscopy, polarizing microscope and thermogravimetric analysis. It was reported that petroleum-derived VRP pitch contained relatively low mesophase (28–41%) of mostly fiber texture and relatively low aromaticity. CTP pitch had highly aromatic structure consisting of polyaromatic molecules rarely substituted by small alkyl substituents. It had 32–54% of mesophase which showed distinct mosaic texture. The CDP pitch consisted of more anisotropic mesophase with a regular and compact lamellar arrangement of the planar polyaromatic molecules with largest average molecular size and condensation degree. It was shown that these features of CDP pitch provided its better graphitizability compared to CTP and VRP pitches.

In our papers [36,37,38,39], polyaromatic quinoline soluble products were obtained by liquid-phase solubilization of coals at mild temperatures using technical heavy hydrocarbon fractions of coal and petroleum origin as solvents. It was found that the yield of quinoline solubles was in the extremal dependence on coal rank [38] and dissolution temperature [39]. The highest yield of quinoline-soluble substances was achieved for bituminous coals in the temperature range of 350–380 °C, when coals passed into a plastic state [38]. The correlation analysis was performed and the coal-related parameters ensuring effective conversion into quinoline-soluble products were determined. The liquid phase solvolysis was shown [37,40] to involve selective depolymerization of the polymer-like coal matter via weakest linkages (ether or/and methylene) between the large polycondenced aromatic nuclei. The soluble products obtained represented solid pitch-like matter consisted primarily of polyaromatic substances [36,37]. It has been demonstrated [41] that the pitch fraction isolated from the coal dissolution products can be used as a binder for the preparation of anodes for aluminum electrolysis.

This study is focused on the comprehensive characterization of chemical and group composition, molecular structure of the pitch-like products obtained by coal thermosolvolysis as possible new feedstock for high-tech carbon materials, needle coke in particular. Bituminous coal is used for dissolution, and the solvents are heavy technical hydrocarbon fractions of coal and petroleum origin. Because of the complex molecular composition, the products obtained are characterized by averaged molecular parameters determined by using a set of analytical techniques, such as ^1^H, ^13^C NMR, FTIR and XPS and liquid-phase chromatography. Particular attention is given to structural features of the aromatic units depending on the type of solvent used. The concentration of benzo(a)pyrene as a marker of carcinogenicity and viscoelastic properties of the products are also characterized.

## 2. Material and Methods

### 2.1. Coal and Solvents Used

Bituminous coal from the Chadan deposit (Ulugkhemsky Basin, Kyzyl, Russia) was used for dissolution. It was ground to a fraction of <1 mm (average particle size of 0.4 mm) and dried in a vacuum oven at 85 °C. The commercial coal tar (CT) (Altai-Coke Joint-Stock Company, Zarinsk, Russia), and heavy gas oil (GO) derived from the catalytic cracking of petroleum fraction (Omsk Oil Refinery Plant, Gazprom-Neft Company, Omsk, Russia) and binary CT + GO blend were used as solvents for coal dissolution. A typical commercially available coal-tar pitch with the softening point of 88 °C provided by Altai-Coke Joint-Stock Company was used as reference sample.

### 2.2. Reactor Unit and Dissolution Procedure

The coal dissolution reaction was carried out following previously optimized conditions [38] using an experimental unit equipped with a 2 L stainless steel autoclave with a stirrer. The autoclave was charged with 900 g of coal/solvent slurry (the proportion of 1:2 by the weight), hermetically sealed and purged carefully with nitrogen. The reaction was carried out at 380 °C for 60 min at an autogenous pressure.

At reaction completion, the autoclave was allowed to cool to 250 °C, then depressurized, and the vapor-gas products were vented through a refrigerator line, while the gases and condensed liquid were collected and measured. The valve at the bottom of the autoclave was then opened, and the molten digested product (consisted of dissolved coal in a solvent + ash coal residue) was drained off into a cylinder receiver, allowed to cool while stirring for homogenization. Then, it was pushed out of the cylinder by a piston. The digested pitch-like product was weighted, immediately packaged in a plastic bag and stored in a desiccator. The gaseous products were analyzed by a gas chromatography.

### 2.3. Analytical Techniques

The elemental analysis was performed using a CHNS-analyzer (Vario EL Cube, Elementar, Langenselbold, Germany). Thermal decomposition of coal was studied using Netzsch Jupiter STA 449F1 (NETZSCH-Gerätebau, Selb, Germany) synchronous analyzer. The volatility of the solvents was determined by temperature range on distillation.

The group composition of the resultant pitch-like products was characterized by the proportions of the toluene solubles (*TS*), quinoline solubles (*QS*), quinoline insolubles (*QIS*, α_1_-fraction) and by α_2_-fraction (*TIS* minus *QIS*). A ground sample of the pitch-like product (weigh of 2 g) was subjected to exhaustive extraction with toluene using a Soxhlet apparatus (Alphapribor, Klin, Russia). Toluene insoluble residue (*TIS*, α-fraction) was dried under vacuum at 80 °C and weighted. Extraction with a hot quinoline was carried out according to the standard method [42]. The pitch-like sample (1 g) was placed in an ampoule where hot quinoline was added. The ampoule was placed in a water bath at temperature of near 75 °C and kept for 30 min, shaking occasionally. Then, the ampoule was centrifuged and treated again with a fresh hot quinoline, the procedure was repeated until the quinoline solution became clear. Finally, the centrifuged quinoline-insoluble residue was washed with toluene, dried in a vacuum at 80 °C and weighed. The proportions of *TS* and *QS* soluble fractions were calculated according to formulas:

(1) $$TS=\frac{m_{0}-m_{TIS}}{m_{0}(1-0.01A_{0})}\times 100$$

(2) $$QS=\frac{m_{0}-m_{QIS}}{m_{0}(1-0.01A_{0})}\times 100$$

where *m*_0_, *m_TIS_*, *m_QIS_* are the weights (g) of the initial pitch sample and of toluene insoluble and quinoline insoluble residues, respectively, and *A*_0_ is ash content (%) in the initial pitch-like product.

The FTIR spectra were recorded using a Bruker Tensor-27 IRFT spectrometer (Bruker Tensor-27 IRFT, Ettlingen, Germany) for the spectral range from 4000 to 400 cm^−1^. The specific spectral regions were subjected to curve-fitting analysis.

The ^1^H and ^13^C NMR high resolution spectra were recorded from the chloroform-D dissolved samples using a Bruker Advance III 300 WB spectrometer (Bruker Tensor-27 IRFT, Ettlingen, Germany) with proton resonance frequency of 300 MHz at a room temperature. For the proton spectrum, 16 scans were accumulated and 1024 scans for the ^13^C spectrum.

The XPS spectra were obtained using a SPECS instrument (SPECS Surface Nano Analysis GmbH, Berlin, Germany) equipped with a PHOIBOS 1500 MCD9 electron analyzer (SPECS Surface Nano Analysis GmbH, Berlin, Germany), with excitation by a Mg Kα line (1253.6 eV) of the X-ray tube and with a charge neutralization gun. Pass energies were 20 eV for survey spectra and 8 eV for high-resolution spectra. The data were collected over 0.05 eV increments, and multiple scans were taken to obtain spectra with high-enough signal-to-noise ratios to allow curve-resolution techniques to be applied. The photoelectron peaks were curve-resolved using a mixed 70–30% Gauss-Lorentzian lines shape to assess the relative amounts of the particular chemical species. A Shirley-type background was subtracted prior to fitting. An energy correction was made to account for sample charging by setting the binding energy of the sp^2^ carbon to 284.6 eV. The atomic concentrations were calculated from the survey spectra as peak areas corrected for atomic sensitivity factors.

The benzo(a)pyrene (BaP) concentration in the initial solvents and in the toluene soluble fractions of the products was measured using Shimadzu LC20 (Shimadzu, Kyoto, Japan) high-performance liquid chromatograph. Chromatography conditions: capillary column with C_18_ reverse phase sorbent, diode array detector, the flow rate of acetonitrile-water mixture was 1 mL/min, the standard BaP solutions were used for calibration.

The viscoelastic properties of the pitch-like products were characterized by softening point using a “ring-and-ball” method [43]. The softening point corresponded to temperature at which a metal ball passed through a ring filled with the softened pitch.

## 3. Results

### 3.1. Characterization of Coal and Solvents

The data in Table 1 show the composition and properties of coal and solvents used. Coal sample was bituminous grade with the volatile matter of 37.8 wt.% (on daf), ash of 5.6 wt.% (on dry bases), softening point of around 350–360 °C, plastic layer thickness of 21 mm, vitrinite content of 85% and vitrinite reflectance coefficient of 0.77%. According to TG/DTG analysis, the thermal decomposition with the weight loss commenced at 400 °C, and maximum rate of weight loss was observed at 465 °C.

Table 1 shows that the petroleum-derived GO solvent was almost completely soluble in toluene, had more hydrogen, less nitrogen, sulfur and very small BaP, compared to CT solvent. The latter contained 11.6% of toluene-insolubles, 1.8% of quinoline-insolubles. Both solvents began to distill off at temperatures above 180–221 °C, the distillation maximum was observed at around 350 °C.

### 3.2. Coal Dissolution

The dissolution of coal at 380 °C and autogenous pressure of near 1.4–2.5 MPa attained 80% and more. The yield of the main digested pitch-like autoclave product (consisted of dissolved coal, solvent and coal ash residue) accounted for at least 97.5%. The yields of gases and naphtha collected during depressurization of the autoclave at 250 °C were less than 0.6% and 0.55 to 4.8%, respectively, depending on the solvent used. The enhanced naphtha yield (4.8%) was obtained when CT was used as solvent. The losses due to difficulty of quantitative recovering of the viscous autoclave product were less than 2.0%.

#### 3.2.1. The Composition of the Pitch-like Products

Shown in Table 2 are characteristics of the products obtained using different solvents. The products obtained using CT and binary solvent represented typical solid pitch-like matter with the softening points of 82 to 90 °C, the GO product was a soft matter without a certain softening point.

The CT product had more carbon and oxygen, and less hydrogen compared to GO product. The products obtained with binary CT + GO blend had intermediate chemical composition compared to those obtained using each solvent separately. The concentrations of nitrogen, sulfur and oxygen were 0.6–1.3%, 0.6–1.4% and 0.8–2.6%, respectively, GO product featured smallest oxygen concentration. The increase in the dissolution duration from 1 h to 3 h resulted in increased carbon content and in reduced hydrogen and oxygen.

The group composition consisted of 64.4 to 77.6% of toluene solubles (maltenes + asphaltenes) and 90.8 to 92.9% of quinoline soluble fractions. GO product showed least and CT product largest amounts of the α_2_-fraction, which contained preasphaltenes and carbenes (Table 3). The content of quinoline insoluble α_1_-fraction ranged 7.1–9.2% depending on the solvent. The CT + GO blended solvent improved coal dissolution compared to each solvent separately: the content of the quinoline insolubles in the product decreased to 7.1% versus 8.2% and 7.9% for the CT and GO products, respectively.

**Figure 1:**
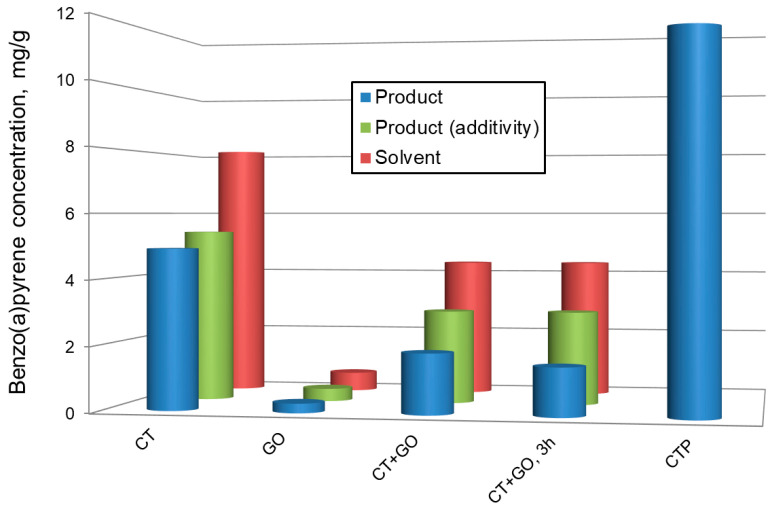
Benzo(a)pyrene content in different solvents and in the products obtained by coal thermosolvolysis in solvents.

The concentration of BaP in the pitch-like products ranged 0.29 to 4.92 mg/g depending on the solvent used (Figure 1). The CT product showed the highest concentration, GO one-the lowest, and that obtained with the blended solvent–an intermediate concentration (in accordance with the BaP concentration in the parent solvents). All the pitch-like products had significantly less BaP (up to 40 times) compared to typical commercial coal-tar pitch (12 mg/g). Two important observations should be noted: (i) the increase in coal dissolution duration (in blended solvent) from 1 to 3 h led to decrease in BaP concentration in the reaction product from 1.84 to 1.48 mg/g; (ii) in all cases, BaP concentrations in the reaction products were significantly less than those in the coal-solvent slurry before reaction. These results indicate BaP to be partially chemically consumed during the coal dissolution, in contrast to coal coking resulting in BaP generation.

#### 3.2.2. Molecular Structure of the Pitch-like Products

**Figure 2:**
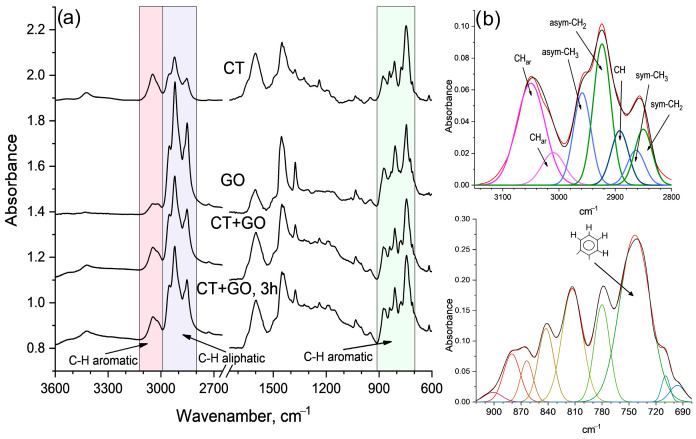
FTIR spectra of the products obtained using different solvents (**a**) and deconvoluted spectra as examples (**b**).

*FTIR spectra*. Figure 2 reflects the variation in the molecular structure of the pitch-like samples. The bands centered at 1600 cm^−1^ (stretching vibrations of the aromatic C-C bonds), 3045 cm^−1^ (stretching vibrations of the aromatic C-H bonds) and at 900–700 cm^−1^ (out-of-plane bending of the aromatic C-H bonds) indicate the aromatic structures. The broad absorbances in the spectral ranges of 3000–2750 cm^−1^ and 1460–1370 cm^−1^ (stretching and bending vibrations of the aliphatic C-H bonds, respectively) indicate the aliphatic structures. Very small absorbance at near 3430 cm^−1^ may indicate small amount of hydrogen-bonded phenols and possibly nitrogen-containing heterocycles with N-H bonds (as in indole and carbazole). Barely visible shoulders at about 1750 cm^−1^ and 1650 cm^−1^ reflect little carboxyl and carbonyl groups.

Absorbance in most characteristic spectral regions of 3100–2750 cm^−1^, and 900–700 cm^−1^ were deconvoluted (Figure 2b) according to well-founded guidelines [44,45]. The corresponding semi-quantitative molecular indexes were assessed based on the deconvoluted spectra. Other spectral regions also show variations in the absorbance depending on the product sample, however, they are less characteristic. For example, the spectra show large variation in the absorbance centered at 1600 cm^−1^. However, the extinction coefficient for stretching vibration of the aromatic C-C bonds depends greatly on the structure of the aromatic unit, type of substituents and degree of substitution. Therefore, this band can hardly be used for semi-quantitative estimation. Complex unresolved bands in the region of 1000–1300 cm^−1^ are related to vibrations of various C-C and C-O bonds. Deconvolution and assignment of individual sub-bands in this region is difficult due to overlapping and also due to superposition of the bands from the silicate and aluminosilicate minerals with Si-O and Al-O bonds.

The absorbance in the 3100–2750 cm^−1^ region was best simulated by two symmetrical Gaussian aromatic sub-bands and by five aliphatic sub-bands. The wide band in the region of 900–700 cm^−1^ was best fitted to 8–10 sub-bands. They reflect the number of adjacent C-H bonds at the aromatic ring, and thus a degree of the aromatic ring substitution (or condensation). A sub-band centered at 740 cm^−1^ was assigned to four adjacent C-H bonds at the aromatic ring (i.e., ortho-substituted ring) [44]. It reflects the degree of the aromatic ring substitution: the higher the intensity, the lower the substitution degree.

The following molecular indexes were estimated based on the areas of the characteristic sub-bands in the deconvoluted FTIR spectra: H_ar_ hydrogen aromaticity; C_ar_ carbon aromaticity; I_os_ index for ortho-substituted aromatic rings; and proportion between the number of C-H bonds in the CH_3_ and CH_2_ groups which reflects the structure of the aliphatic fragments (the length or branching of alkyl groups).

In assessing H_ar_, C_ar_ and CH_3_/CH_2_ indexes we took account of the corresponding extinction coefficients. According to [44,46], the ratio of the extinction coefficient for the stretching vibrations of the aromatic C-H bonds (3100–3000 cm^−1^) to that for the aliphatic C-H bonds (3000–2750 cm^−1^) was accepted to be 0.20, and that for stretching vibrations of C-H bond in the CH_2_ groups (sub-band centered at 2923 cm^−1^) and in the CH_3_ groups (sub-band centered at 2955 cm^−1^)–0.5. C_ar_ index was estimated according to [47] using the ultimate analysis data and FTIR data for the proportion of the aliphatic hydrogens to total amount of hydrogens in an average molecule. The semi-quantitative molecular indexes above were calculated using the following formulas:

(3) $$H_{ar}=\frac{A_{ar}/0.2A_{al}}{1+\left(A_{ar}/0.2A_{al}\right)}$$

(4) $$C_{ar}=1-\frac{C_{al}}{C};\;\;\frac{C_{al}}{C}=\frac{\left(\frac{H_{al}}{H}\times \frac{H}{C}\right)}{\left(\frac{H_{al}}{C_{al}}\right)}$$

(5) $$I_{os}=\frac{A_{750}}{A_{900-700}}$$

(6) $$\frac{CH_{3}}{CH_{2}}=0.5\times \left(\frac{A_{2955}}{A_{2923}}\right)$$

*H_al_*/*C_al_* in Equation (4) is an atomic ratio of hydrogen to carbon for aliphatic groups, usually set to 2 [47].

One can see from Table 4 that the FTIR indexes show highly aromatic structure of the CT product (*C_ar_* = 0.87 and *H_ar_* = 0.67). Its aromatic rings are rarely substituted (ortho-substituted) mostly with methyl and methylene groups. GO product is least aromatic (*C_ar_* = 0.64 and *H_ar_* = 0.31), the aromatic rings being highly substituted (*I_os_* = 0.20) with larger alkyl substituents (*CH_3_/CH_2_* ratio of 0.33). The product obtained with binary solvent shows molecular indexes which are almost intermediate compared to those obtained with each solvent separately. Increasing the dissolution time (by 3 h) results in increased aromaticity, CH_3_/CH_2_ ratio and in decreased aromatic ring substitution.

**Figure 3:**
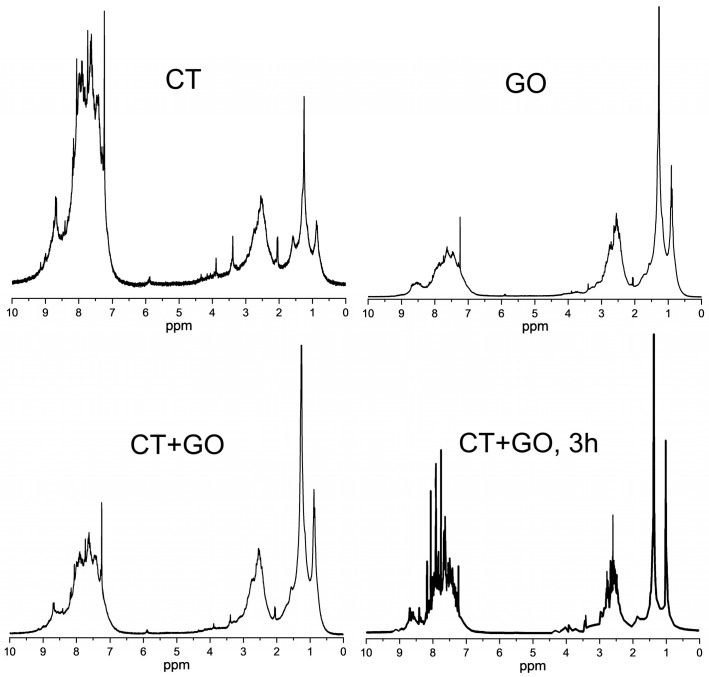
The ^1^H NMR spectra of the chloroform-solubles derived from the products obtained using CT, GO solvents and CT + GO solvent blend.

*^1^H NMR spectra*. The spectral resonances in Figure 3 were subdivided according to [48,49] into the following groups: 9.5–6.7 ppm due to aromatic protons (H_ar_), 6.7–4.5 ppm–to H_o_ protons in the olefinic and hydroxyl groups; 4.5–3.4 ppm–to H_α1_ protons at the α-carbon bonded to two aromatic rings; 3.4–2.0 ppm–to H_α2_ protons at other α-carbons; 2.0–1.0 ppm–to H_β_ protons at saturated carbons in β-position to the aromatic ring; 1.0–0.5 ppm–to H_γ_ protons at carbons in γ-position and others.

The spectrum from the CT product shows strong resonances in the aromatic region and very weak resonances in the aliphatic one. Most aliphatic protons are bonded to carbon atoms at α-position to one aromatic cycle (3.4–2.0 ppm) and also to two cycles (like in fluorene or in 9,10-dihydroanthracene, 4.5–3.4 ppm). The products obtained with GO solvent and with blended one are less aromatic, they show large variety of protons at various aliphatic carbon atoms, including those at α-position to aromatic rings.

The integrated data on the proton distribution and Brown-Ladner structural parameters calculated are shown in Table 5. The average molecules of the chloroform-solubles derived from the CT product are highly aromatic (*H_ar_* of 0.65 and f_a_ of 0.85), closely resembling typical coal-tar pitch. The H_aru_/C_ar_ index means the aromatic nuclei to consist of 4–5 condensed cycles, which are rarely substituted (σ = 0.16) with short alkyl substituents (n = 1.8). The aromatic nuclei of the GO product consist of 3–4 highly substituted (σ = 0.37) condensed cycles with longer substituents (n = 2.6). The CT + GO product is intermediate in its indexes compared to CT and GO products. The ^1^H NMR spectra show insignificant concentrations of the hydroxyl groups and olefinic substances in all samples.

Displayed in Table 5 are also the structural characteristics of the parent CT and GO solvents for comparison. It can be noted that the CT pitch-like product, compared to parent CT solvent, is characterized by a reduced aromaticity (f_a_ of 0.88 versus 0.95), however, by higher degree of the aromatic ring condensation (H_aru_/C_ar_ of 0.63 versus 0.68). The GO product, compared to GO solvent used, features higher aromaticity (0.64 versus 0.60) and also higher condensation degree (0.68 versus 0.79).

*^13^C NMR spectra*. While ^1^H NMR spectra reflect the composition of the periphery of the aromatic clusters, ^13^C NMR spectra provide also an information related to the internal carbon framework.

**Figure 4:**
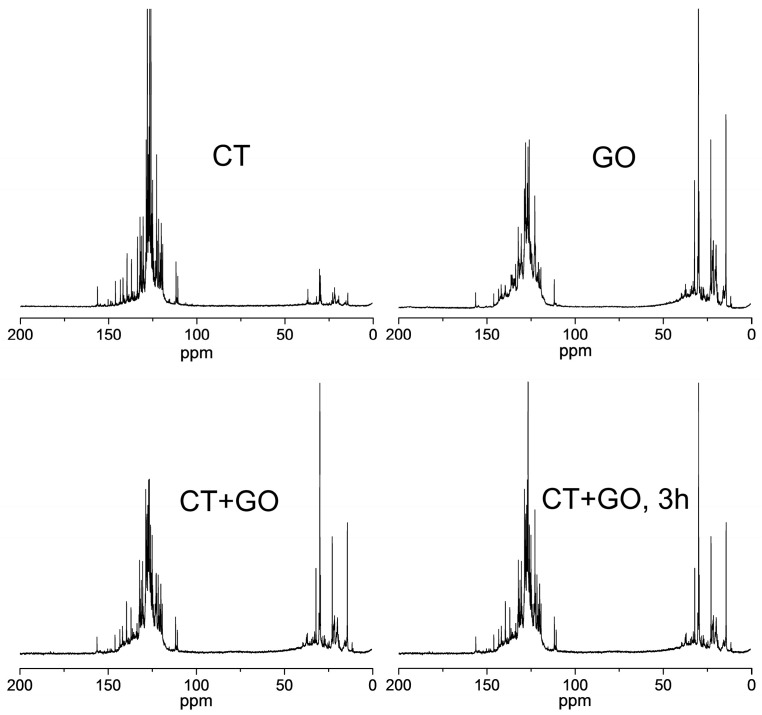
The ^13^C NMR spectra from the chloroform-D solubles of the products obtained using different solvents.

The high-resolution ^13^C NMR spectra from the chloroform-D soluble fractions are displayed in Figure 4. According to [50,51,52], the spectra were subdivided into the following sub-regions: 0–24 ppm related to CH_3_; 24–49.3 ppm–to CH_2_ and CH groups including those in α-position to aromatic cycles; 55–70 ppm–to aliphatic C-O; 108–129.5 ppm–to quaternary pericondensed aromatic carbon, i.e., to carbon atom belonging to three aromatic rings (C_ar3_) and also to tertiary protonated aromatic carbon (C_ar_H); 129.5–155 ppm–to quaternary catacondensed carbon, i.e., to carbon belonging to two aromatic rings (C_ar2_) and to quaternary aromatic carbon bonded to aliphatic carbon (C_ar_C); 155–165 ppm–to C_ar_O; 165–187 ppm–to COOH; 187–220 ppm–to C=O.

One can see from Figure 4 that all the spectra show resonances from the aromatic carbons to be well separated from the aliphatic carbons. The contribution from the oxidized carbon groups (COO and C=O, sub-region above 155 ppm) are small in all the products. Displayed in Table 6 are the normalized integration ^13^C NMR data on the distribution of carbon atoms between different groups. The f_a_ aromaticity was determined as the sum of (C_ar3_ + C_ar_H) + (C_ar2_ + C_ar_C) + (C_ar_O). The obtained molecular structural characteristics are in good agreement with the characteristics obtained from FTIR (Table 4) and from ^1^H NMR spectra (Table 5). They corroborate highly aromatic nature of the CT product (f_a_ = 0.87) with rarely substituted aromatic nuclei containing predominantly pericondensed + protonated cycles (C_ar3_ + C_ar_H). The product obtained using GO solvent shows lower aromaticity (f_a_ = 0.64) and proportion of C_ar3_ + C_ar_H groups, while the proportion of C_ar2_ + C_ar_C groups is almost same as in the case of the CT product.

The aliphatic structure of the products is represented by CH_3_ and CH_2_ + CH groups, the GO product showing large variety of aliphatic groups including those at α-positions to aromatic rings. The amounts of carbon-oxygen groups are small in all samples.

The ratio between peri- and catacondensed structures is an important characteristic of the structure of the aromatic nuclei in molecules for the production of carbon materials, needle coke, in particular. The ^13^C NMR technique does not allow determining the proportions between C_ar3_ and C_ar_H and between C_ar2_ and C_ar_C, since the signals from the C_ar3_ and C_ar2_ aromatic carbon atoms overlap with the signals from the corresponding peripheral carbon atoms (C_ar_H and C_ar_C, respectively). However, it is possible to estimate the C_ar_H value from the H_ar_ value (estimated from the FTIR spectra), recalculated for carbon taking account of H/C ratio. By subtracting the C_ar_H value thus estimated from the total content of C_ar3_ + C_ar_H groups, one can determine the proportion of carbon atoms in the pericondensed C_ar3_ cycles.

The data in Table 6 show the aromatic nuclei in all the products to contain mainly protonated carbon atoms (47 to 55% of all the aromatic carbon atoms). The C_ar3_ fraction is the largest in the CT product (28% of all the aromatic carbon atoms) and the smallest in the GO one (19%). The product obtained with binary solvent has average structural indexes. Increasing the dissolution duration (from 1 to 3 h) results in increase in the proportion of carbon atoms in the pericondensed cycles due to protonated ones.

**Figure 5:**
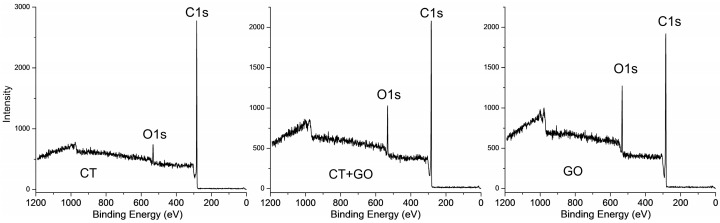
The typical X-ray photoelectron survey spectra for the products obtained using different solvents.

*XPS spectra*. The survey photoelectron spectra in Figure 5 show a major peak at near 285 eV attributed to C1s and much weaker peak at near 533 eV related to O1s. Very weak peak of N1s (near 400 eV) was also observed indicating minor concentrations of the nitrogen-containing species in all samples. Trace amounts of inorganic elements such as Si, Al, Ca, Fe and S can also be found on the surface.

The concentrations of the elements on the surface of the products were estimated based on the survey spectra. Shown in Table 7 are the atomic surface concentrations of the main elements normalized to C1s + O1s + N1s + Si2p + Ca2p = 100%. The concentrations of carbon and oxygen range 85.6 to 89.9% and 7.2 to 13.5%, respectively, depending on the sample. GO product has more oxygen concentration compared to CT product and the product obtained using blended solvent does intermediate concentrations. Actually, the data on O/C atomic ratios in Table 7 mean, that the surface of all the pitch-like samples are enriched with oxygen. At the same time, nitrogen distribution between the surface and the bulk varies moderately and almost equally in all the products. The fact that the GO product exhibits highest concentration of surface oxygen and the CT product the lowest seems to be surprising and contrasts with the bulk oxygen concentrations (Table 2).

The surface enrichment with oxygen could not result from the oxide minerals, since they were coarsely dispersed, and the contents were equal and small (about 2%) in all the products. Thus, the phenomenon of surface enrichment with oxygen resulted most likely from the oxidation of surface carbons upon contact with the atmospheric oxygen when the samples were stored (in a desiccator) for 8–10 days prior XPS analysis. However, no changes in the surface concentration were detected in GO and CT samples stored for longer periods (up to 6 months).

*C1s spectra*. The binding energy of C1s bonded to oxygen differs significantly depending on whether it is bonded to one oxygen atom by a single bond, by a double bond, or bonded to two oxygen atoms. High-resolution XPS O1s spectra were also analyzed. However, the data obtained were less reliable due to relatively low sensitivity and presence of inorganic oxides.

**Figure 6:**
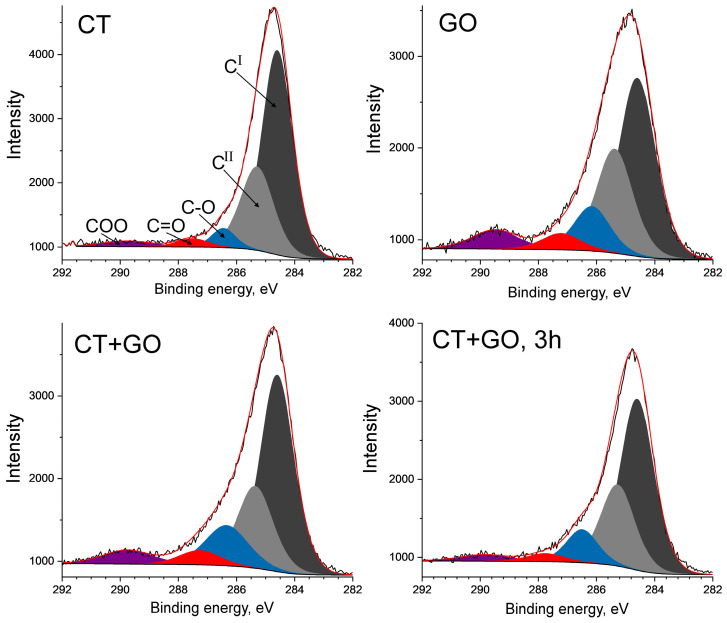
C1s XPS from the products obtained using CT, HGOCC solvents and their binary blend.

Displayed in Figure 6 are the C1s XPS spectra, which show broadened C1s peaks with an asymmetry towards a higher binding energy due to oxygen effect. In all cases, the C1s peak was best fitted to five symmetrical Gaussian/Lorentzian sub-peaks with the binding energies of 284.6 eV, 285.2 eV, 286.3 eV, 287.8 eV and 289.5 eV. The major sub-peak centered at binding energy of 284.6 ± 0.2 eV was assigned to sp^2^-hybridized carbons in the polycondensed aromatic clusters or in graphite-like species (C^I^ component) [53,54]. The sub-peak at 285.2 ± 0.2 eV was assigned to other sp^2^-hybridized aromatic carbons (possibly, in small cycles) as well as to sp^3^-hybridized aliphatic carbons (C^II^ component). Three other sub-peaks at higher binding energies were assigned to oxidized carbon atoms: at 286.3 ± 0.2 eV to C-O (hydroxyls/ethers/epoxides), at 287.8± 0.2 eV to C=O (carbonyls, ketones and quinones), at 289.5 eV to COOH and COO (acids and esters, respectively). Figure 6 shows that the graphite-like C^I^ component dominates the deconvoluted spectra.

Summarized in Table 8 are the distribution of the surface carbon atoms between the particular chemical forms. The cumulative content of the aromatic and aliphatic carbons (C^I^ + C^II^) ranges 0.72 to 0.88, with the proportion of the major C^I^ carbons varying from 0.44 to 0.60, and that of C^II^ hardly changing (0.26–0.28). The total proportion of the oxidized carbon ranges 0.12 to 0.28, with the largest share corresponding to GO product, the smallest to CT product, and to commercial coal-tar pitch. The latter, by the way, was stored under atmospheric conditions during a long period. The products obtained with the binary CT + GO solvent show a nearly intermediate proportion of oxidized carbon compared to the products obtained with each solvent separately. The increase in the dissolution time (3 h) results in some reduction in carbon oxidation. Single bonded surface carbon-oxygen groups such as ethers, phenols and epoxides predominate in all the products.

It should be noted that the major C^I^ sub-peak varied significantly with full width at half maximum (FWHM) depending on the sample (Table 8): it was widest for the GO product (1.54 eV) and narrowest for the CT one (1.25 eV), like for a commercial coal-tar pitch (1.23 eV). The peak broadening could be due to a number of factors: to the extent of surface homogeneity, to size of polycondensed clusters and their structural orientation, and also to presence of heteroatoms on the surface, oxygen, in particular. Mateos et al. [54] reported that the presence of oxygen on the carbon surface can induce the structural defects resulting in peak broadening. In fact, a relation between the FWHM and the surface oxygen concentration is observed in Table 8: the higher the surface oxygen concentration, the wider the C^I^ sub-peak. Thus, the broadened resonance from the C^I^ carbon atoms for the GO product may reflect its defected surface structure. And the narrower resonance from the CT product may indicate its more regular surface structure consisted of mainly protonated and pericondensed aromatic cycles.

## 4. Discussion

The data obtained show that the liquid-phase reaction of bituminous coal with the coal tar and heavy petroleum gas oil as solvents at moderate temperature of 380 °C allows for deep and selective conversion of the organic coal matter into quinoline-soluble substances. A binary blend of highly aromatic CT solvent with GO having more aliphatic nature shows some synergistic effect resulting in less amount of quinoline insolubles (7.1%) compared to both CT (8.2%) and GO (7.9%) solvents separately. The major product with the yields of at least 97.5% represented pitch-like matter with the softening points of 82 to 90 °C, like a typical coal-tar pitch. The distillation of the pitch-like products yielded 14.7–18.7% of liquid fractions with the boiling point of below 350 °C and 81.3–85.3% of pitch residues.

The pitch-like products obtained using CT solvent represented highly aromatic matter (carbon aromaticity of 0.87–0.88 and hydrogen aromaticity of 0.64–0.67). The aromatic nuclei of the chloroform soluble substances contained 4–5 predominantly protonated and pericondensed rings rarely substituted mainly with CH_3_ and occasionally CH_2_ groups. The pericondensed rings contained about 28% of all aromatic carbon atoms. The product obtained with GO solvent showed relatively low aromaticity, its aromatic nuclei contained mainly protonated and highly alkylated catacondensed chains, rather than pericondensed sheets. The intermediate structural parameters were characteristic of the pitch-like product obtained using binary solvent.

According to XPS spectra, the pitch-like products are prone to autogenous surface oxidation. The GO product, which has relatively low aromaticity and highly substituted aromatic rings, is most susceptible to surface oxidation. The oxidized surface carbon species were represented mainly by single-bonded C-O groups (phenols, ethers, and epoxides) in all samples.

The air-blowing oxidation at a moderate temperature is conventionally used to improve pitch properties [55,56,57]: to increase C/H ratio, softening point, content of carbon residue, toluene insoluble fraction, and to control the thermoplastic properties, mesophase formation, and pitch expansion on carbonization. The reactivity for oxidation has been shown [57,58] to depend on the pitch structure. Alkyl-substituted and catacondensed aromatics are easily oxidized, the benzylic carbon atoms are the most reactive and susceptible to oxygen attack. This is consistent with high tendency for surface oxidation of GO product containing highly substituted catacondensed aromatic rings.

A remarkable feature of the products obtained is reduced content of BaP. The product obtained using petroleum-derived solvent had 40 times less BaP than typical coal tar pitch. There are two interesting points to note: (i) unlike high temperature coking, coal dissolution did not generate BaP, and (ii) moreover, BaP introduced into the reaction mixture with the solvent undergoes chemical conversion during coal dissolution.

The data obtained contribute to more insight into molecular structure of the pitch-like products obtained by low-temperature coal dissolution, which, in turn, is important for a better understanding of their properties and predicting rational application. It can be assumed based on the composition that the pitch like products can serve as a new polyaromatic feedstock with a reduced carcinogenicity for the production of valuable high-tech carbon materials. The CT product, in particular, can serve as a preferred feedstock for the production of needle coke. It has a high concentration of rarely substituted polyaromatic mesogens with pericondensed rings. Compared with commercial coal tar, its chemical composition has a reduced nitrogen and sulfur concentration, and polyaromatic mesogens are characterized by a higher condensation degree, which favors [57] the formation of large anisotropic mesophase domains-the precursors of needle coke.

On the other hand, the GO product, which is characterized by protonated and catacondensed highly substituted aromatic units can serve as a feedstock for carbon fiber. Compared with commercial heavy gas oil, it has higher aromaticity and degree of aromatic ring condensation, as well as increased reactivity for oxidation to adjust properties.

In total, by selecting solvents for coal dissolution and by using product fractionation, it is possible to optimize the molecular-structural characteristics of the dissolved products in order to obtain most favorable feedstock. The authors intend to conduct an experimental study of the process of delayed coking of various pitch-like products and to investigate the properties of coke products.

## 5. Conclusions

- The liquid-phase reaction of bituminous coal with commercially available heavy hydrocarbon fractions of coal- and petroleum origin at moderate temperature of 380 °C and autogenous pressure of 1.4 to 2.5 MPa with no catalyst and hydrogen resulted in deep and selective coal dissolution into quinoline-soluble substances (to more than 80%), the yield of gaseous products being no more than 0.5%. The binary blend of highly aromatic coal tar and aliphatic petroleum-derived heavy gasoil fraction exhibited some synergistic effect resulting in improved coal dissolution.
- The resultant products represented typical pitch-like matter with the softening points of 82 to 90 °C. Comprehensive characterization by FTIR, ^1^H NMR, ^13^C NMR, XPS spectroscopy and liquid phase chromatography showed the products to consist of predominantly polycondensed aromatics, which structural parameters strongly depended on the solvent type.
- The product obtained using coal tar as solvent had highly developed aromatic structure, its polycondensed nuclei consisted of predominantly protonated and pericondensed cycles sparsely substituted by CH_3_ and occasionally CH_2_ groups. The product obtained using petroleum-derived solvent was less aromatic, its aromatic nuclei contained protonated and highly alkylated catacondensed chains. The intermediate structural parameters were characteristic of the product obtained using binary solvent.
- All the pitch-like products obtained had a reduced BaP concentration, the smallest concentration showing the product obtained using petroleum-derived solvent (40 times less than in typical coal-tar pitch). An increase in coal dissolution duration further reduced BaP concentration.
- The product obtained using petroleum-derived solvent was prone to autogenous surface oxidation by the atmospheric oxygen at room temperature. The surface of the product obtained using coal tar was much less oxidized, just like a commercial coal-tar pitch sample.
- In terms of molecular composition, the pitch-like products obtained by low-temperature dissolution of coal can serve as polyaromatic feedstock with a reduced carcinogenicity for the production of valuable carbon materials. By selecting solvents, it is possible to optimize the molecular-structural characteristics of the dissolved products in order to obtain favorable feedstock.
- The CT product with highly developed polycondensed aromatic structure can serve as a preferred feedstock for the production of needle coke. The GO product, which is characterized by catacondensed and highly substituted aromatic units, can serve as a feedstock for carbon fiber. Its increased oxidation ability contributes to production of pitch with controlled viscoelastic properties for fiber spinning.

We anticipate the success of obtaining pitch-like products by liquid-phase thermosolvolysis of coal under mild conditions without catalyst and hydrogen to be an important key to innovative development of the production of new feedstock for various valuable carbon materials.

## Figures and Tables

**Table 1:** The characterization of coal and solvents used.

Coal, Solvent	Element Composition, wt.%						BaP Content, (mg/g)	Distillation Temperature Range, °C	Toluene Insolubles, wt.%	Quinoline Insolubles, wt.%
	C	H	N	S	O	H/C at.				
Coal	84.7 *	5.5 *	1.3 *	0.6 *	7.9 *	0.78	-**	–	–	–
CT	91.5	5.3	1.6	1.4	0.2	0.69	8.1	180–550	11.6	1.8
GO	89.9	8.3	0.3	0.8	0.7	1.11	0.59	221–508	0.1	<0.1

* based on daf coal. ** no data.

**Table 2:** Chemical composition of pitch-like products obtained on coal dissolution with different solvents for 1 h.

Solvent Used	Content, wt.% Based on daf					H/C	Softening
	C	H	N	S	O	atom.	Point, °C
CT	89.7	5.4	1.3	1.0	2.6	0.72	86
GO	90.3	7.4	0.6	0.9	0.8	0.98	-
CT + GO	88.7	6.6	1.1	1.3	2.3	0.89	82
CT + GO, 3 h *	89.7	5.9	1.2	1.4	1.8	0.79	90
Commercial coal tar pitch	92.5	4.6	1.1	0.6	1.2	0.60	88

* coal dissolution duration was 3 h.

**Table 3:** The group composition of the products obtained on coal dissolution using different solvents.

Solvent Used	Group Composition, wt.% Based on daf Product			
	TS	QS	QIS (α_1_-Fraction)	QS-TIS (α_2_-Fraction)
CT	64.4	91.8	8.2	27.4
GO	77.6	92.1	7.9	14.5
CT + GO	73.0	92.9	7.1	19.9
CT + GO, 3 h	71.0	90.8	9.2	19.8
Commercial coal tar pitch	64.9	89.5	10.5	24.6

**Table 4:** The characterization of molecular composition of the products based on the FTIR spectra.

Solvent Used	Aromaticity Index		Ortho-Substitution, I_os_	CH_3_/CH_2_
	C_ar_	H_ar_		
CT	0.87	0.67	0.44	0.42
GO	0.64	0.31	0.20	0.33
CT + GO	0.76	0.46	0.35	0.34
CT + GO, 3 h	0.78	0.50	0.37	0.41

**Table 5:** Proton distribution and Brown-Ladner structural parameters of the average molecules of the parent solvents and pitch-like products obtained by coal dissolution.

Sample	Proton Distribution					Brown-Ladner Parameters *			
	H_ar_	H_o_	H_α_	H_β_	H_γ_	f_a_	H_aru_/C_ar_	*σ*	n
*Pitch-like product, solvent used*									
CT	0.65	0.02	0.18	0.12	0.03	0.88	0.63	0.16	1.8
GO	0.30	0.01	0.28	0.34	0.07	0.64	0.68	0.37	2.6
CT + GO	0.38	0.01	0.23	0.28	0.10	0.73	0.64	0.26	2.5
CT + GO, 3 h	0.44	0.002	0.22	0.27	0.07	0.78	0.58	0.23	2.5
Commercial coal tar pitch	0.67	0.02	0.15	0.13	0.03	0.91	0.50	0.12	2.0
*Parent solvent*									
CT	0.85	0.01	0.13	0.01	0.001	0.95	0.68	0.11	1.1
GO	0.28	0.004	0.29	0.34	0.09	0.60	0.79	0.35	2.5

* f_a_—carbon aromaticity; σ—degree of aromatic ring substitution; n—average number of carbon atoms in the substituents; H_aru_/C_ar_—hydrogen to carbon ratio in the hypothetical unsubstituted aromatic ring, indicates degree of the aromatic ring condensation.

**Table 6:** Carbon distribution in the products obtained using different solvents.

Solvent Used	CH_3_	CH_2_+ CH	OCH_3_	COC	C_ar_O	C=O + COOH	C_ar3_+ C_ar_H	Including		C_ar2_+ C_ar_C	f_a_
								C_ar3_	C_ar_H *		
CT	0.04	0.06	0.01	0.02	0.01	0	0.71	0.24	0.48	0.15	0.87
GO	0.13	0.15	0.02	0.04	0.04	0.02	0.42	0.12	0.30	0.18	0.64
CT + GO	0.09	0.10	0.01	0.02	0.01	0.01	0.59	0.18	0.41	0.17	0.77
CT + GO, 3 h	0.08	0.11	0.003	0.01	0.01	0.01	0.61	0.21	0.40	0.17	0.79

C_ar3_—quaternary pericondensed aromatic carbons; C_ar_H—tertiary protonated aromatic carbons; C_ar_C—alkylated aromatic carbons; C_ar2_—quaternary catacondensed aromatic carbons. * Derived from the FTIR spectra and chemical analysis.

**Table 7:** The concentrations of the elements on the surface of the products, and the oxygen and nitrogen distribution between the surface and the bulk.

Solvent Used	Surface Concentration, % at					O/C Atomic		N/C Atomic	
	C	O	N	Si	Ca	Surface	Bulk	Surface	Bulk
CT	89.9	7.2	2.0	0.4	0.5	0.06	0.022	0.019	0.012
GO	85.6	13.5	0.6	0.2	0.1	0.12	0.007	0.006	0.006
CT + GO	87.2	9.9	1.4	0.3	0.2	0.08	0.019	0.014	0.011
Commercial coal-tar pitch	92.3	5.5	2.2	-	-	0.04	0.010	0.20	0.010

**Table 8:** The distribution of carbon atoms between different carbon-containing species on the surface of the products obtained using different solvents.

Solvent Used	Non-Oxidized Carbon			Oxidized Carbon				FWHM for C^I^, eV
	C^I^	C^II^	Total	C-O	C=O	COOH	Total	
CT	0.60	0.28	0.88	0.06	0.03	0.03	0.12	1.25
GO	0.44	0.28	0.72	0.14	0.08	0.06	0.28	1.54
CT + GO	0.53	0.27	0.80	0.11	0.04	0.05	0.20	1.37
CT + GO, 3 h	0.55	0.28	0.83	0.11	0.03	0.03	0.17	1.35
Commercial coal tar pitch	0.74	0.16	0.90	0.07	0.01	0.02	0.10	1.23

## Data Availability

The original contributions presented in this study are included in the article. Further inquiries can be directed to the corresponding authors.
